# A *Cryptosporidium parvum* vaccine candidate effect on immunohistochemical profiling of CD4^+^, CD8^+^, Caspase-3 and NF-κB in mice

**DOI:** 10.1186/s12917-023-03699-w

**Published:** 2023-10-19

**Authors:** Dina Aboelsoued, Nagwa I. Toaleb, Sally Ibrahim, Raafat M. Shaapan, Kadria N. Abdel Megeed

**Affiliations:** 1 Department of Parasitology and Animal Diseases, Veterinary Research Institute, National Research Centre, El Buhouth Street, Dokki, Cairo Egypt; 2 Department of Animal Reproduction and AI, Veterinary Research Institute, National Research Centre, El Buhouth Street, Dokki, Cairo Egypt; 3 Department of Zoonotic Diseases, Veterinary Research Institute, National Research Centre, P.O. 12622, El Buhouth Street, Dokki, Cairo Egypt

**Keywords:** *Cryptosporidium parvum*, Vaccine, CD4^+^, CD8^+^, Caspase-3, NF-κB, Immunohistochemistry

## Abstract

**Background:**

*Cryptosporidium parvum* is a protozoan parasite of medical and veterinary importance that causes neonatal diarrhea in many vertebrate hosts. In this study, we evaluated the efficacy of an affinity-purified antigen as a *C. parvum* vaccine candidate using ileal and liver tissues of experimentally infected neonatal mice by immunohistochemical profiling and immune scoring of CD4^+^, CD8^+^, Caspase-3, and nuclear factor kappa B (NF-κB). This vaccine was prepared from the *C. parvum* oocysts antigen using immune affinity chromatography with cyanogen bromide-activated Sepharose-4B beads.

**Methods:**

Thirty neonatal mice were divided into three groups (10 mice/group): (1) non-immunized non-infected, (2) non-immunized infected (using gastric tubes with a single dose of 1 × 10^5^ of *C. parvum* oocysts in 250 µl PBS solution 1 h before a meal) and (3) immunized (twice with 40 µg/kg of purified *C. parvum* antigen at 2-week intervals and then infected with 1 × 10^5^ *C. parvum* oocysts simultaneously with the second group). After euthanizing the animals on the 10th day, post-infection, their ileal and liver tissues were collected and prepared for immunohistochemistry (IHC) staining to detect CD4^+^, CD8+, Caspase-3, and NF-κB levels, which are indicators for T helper cells, cytotoxic T cells, apoptosis, and inflammation, respectively.

**Results:**

The IHC results showed that CD4^+^, CD8^+^, Caspase-3, and NF-κB expression varied significantly (*P <* 0.001) in both organs in all the groups. We also recorded high CD4^+^ levels and low CD8^+^ expression in the non-immunized non-infected mice tissues, while the opposite was observed in the non-immunized infected mice tissues. In the immunized infected mice, the CD4^+^ level was higher than CD8 + in both organs. While the Caspase-3 levels were higher in the ileal tissue of non-immunized infected than immunized infected mice ileal tissues, the reverse was seen in the liver tissues of both groups. Furthermore, NF-κB expression was higher in the liver tissues of non-immunized infected mice than in immunized infected mice tissues. Therefore, the IHC results and immune-scoring program revealed a significant difference (*P <* 0.001) in the CD4^+^, CD8+, Caspase-3, and NF-κB expression levels in both ileal and liver tissues of all mice groups, which might be necessary for immunomodulation in these tissues.

**Conclusions:**

The improvement observed in the immunized infected mice suggests that this vaccine candidate might protect against cryptosporidiosis.

## Background

*Cryptosporidium parvum* (*C. parvum*) is a ubiquitous apicomplexan parasite that mainly infects the gastrointestinal epithelium of humans and animals [1, 2]. It mainly inhabits the apical part of the epithelial cells and does not cause systemic infection or deep-tissue penetration [3, 4]. Additionally, cryptosporidiosis might extend into the biliary epithelium. As the parasite uses the host’s biological processes for its replication [5], the host’s epithelial cells are essential for its survival and protective immune responses [6].

Although the cell-mediated immune response to cryptosporidiosis is known to be via CD4^+^ T cells, the role of CD8^+^ T cells is not yet clear [7, 8]. Moreover, CD4^+^ T cells are important for the immune response to *Cryptosporidium* infection in both human and murine models [9]. CD4^+^ T cells and interferon-gamma (INF-γ) were found to be the dominant immune agents against murine *C. parvum* infection as the IFN-γ produced by these cells is required for infection clearance [9, 10]. Studies suggested that CD8^+^ T cells might contribute to the cell-mediated immune response against *Cryptosporidium* via direct cytolysis of the infected intestinal epithelial cells along with IFN-γ-mediated protection and clearance [11, 12]. Leav et al. [13] also demonstrated that CD8^+^ T cells are involved in early innate immunity in newborn mice.

As the intestinal mucosa is exposed to environmental antigens and pathogens, it contains multiple populations of innate and adaptive immune cells, such as the memory CD4^+^ T cells found throughout the organized intestinal lymphoid tissues, that contribute to many immune functions [14]. Additionally, CD4^+^ T cells are predominantly found location at the lymphoid tissues of the body, and they outnumber CD8^+^ T cells in mucosal tissues and barrier surfaces [15, 16]. In addition to their role in protective responses, they mediate potential in situ immune responses after collateral tissue damage, causing tissue-specific inflammatory disease. Therefore, the contribution of tissue-resident memory T-cell responses should be considered [17]. Host cell death after parasitic infection is represented by the tissue damage resulting from an immediate defense response against intracellular pathogens, such as *C. parvum* [18, 19]. During apoptosis, the cellular contents are packaged into apoptotic bodies, which are phagocytosed by other cells without a robust inflammatory response [20]. The key morphological changes of this process, mediated by caspases are irreversible [21]. Thus, Caspase activity must be regulated to prevent undesired host cell death [21, 22].

Parasites, including protozoans and helminthes, have developed evolutionary strategies that can interfere with cell death to survive and multiply inside the host tissues [23]. Many studies suggested that *C. parvum* has multiple strategies to control apoptosis to facilitate its growth and life cycle completion. Within a few hours of infection, *C. parvum* activates nuclear factor kappa B (NF-κB), which, in turn, activates the antiapoptotic mechanisms [24–26]. It has also been reported to limit host cell death [18]. During the later stages of infection, these antiapoptotic mechanisms disappear, causing host cell death [27]. The inhibition and induction of epithelial cell apoptosis are the underlying basis of the host-parasite interaction [8]. In vitro infection of human tumor cell lines showed that *C. parvum* only replicates in cells in which apoptosis is inhibited [25] due to NF-κB activation [24, 28]. However, the key components involved in the rescue from apoptosis might differ depending on the type of cells and parasites [29]. The invasion of intestinal and biliary epithelium by *C. parvum-*activated TLR4/MyD88/NF-κB signaling, in vitro, triggers the secretion of various cytokines and chemokines that can kill the parasite or inhibit its growth and induce the expression of pro- and anti-apoptotic factors [30].

Here, we evaluated an affinity-purified antigen as a vaccine candidate against *C. parvum* in experimentally infected neonatal mice. The efficacy of this vaccine was assessed using immunohistochemical (IHC) tests and immune scoring of CD4^+^, CD8^+^, Caspase-3, and NF-κB in the examined intestinal and liver tissues.

## Results

### IHC staining

**Fig. 1:**
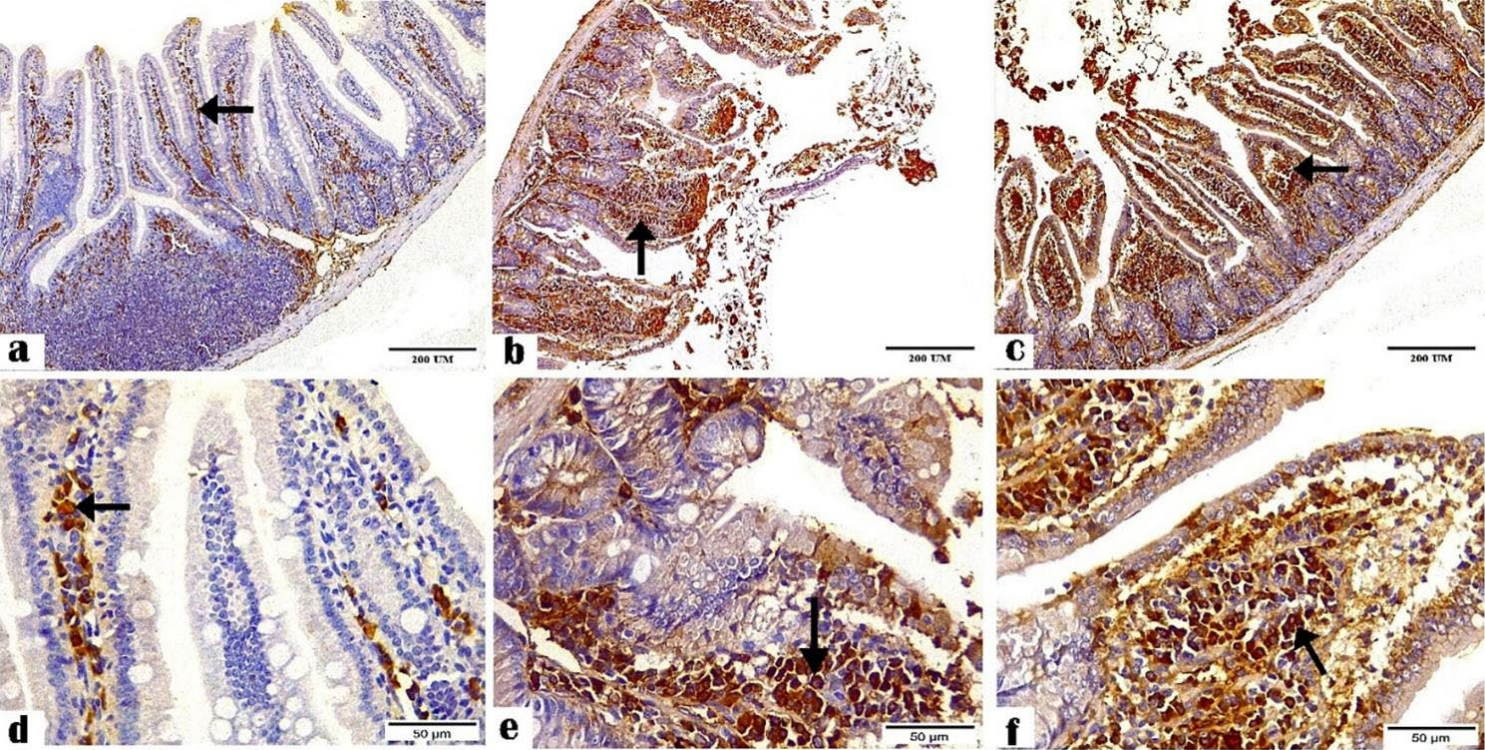
Photomicrographs showing CD4^+^ antibody staining in the ileal tissue sections of the (**a and d**) negative control, (**b and e**) *C. parvum*-infected, and (**c and f**) immunized infected groups indicating mild, moderate, and high CD4^+^ expression, respectively (arrows). (CD4^+^ antibody, magnification: 100 × and 400 ×, respectively, scale bar: 200 and 50 μm, respectively)

**Fig. 2:**
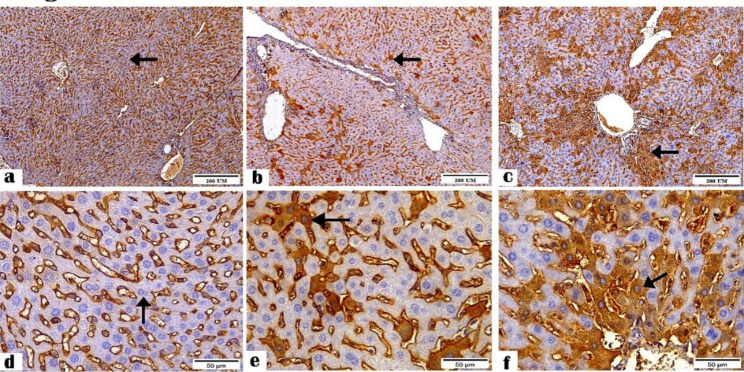
Photomicrographs showing CD4 + antibody reactivity in the liver tissue sections of (**a and d**) the negative control, (**b and e**) *C. parvum*-infected, and (**c and f**) immunized infected groups indicating mild, moderate, and highest reactivity to CD4^+^ antibody, respectively (arrows). (CD4^+^ Antibody, magnification: 100 × and 400 ×, respectively, Scale bar: 200 and 50 μm, respectively)

**Fig. 3:**
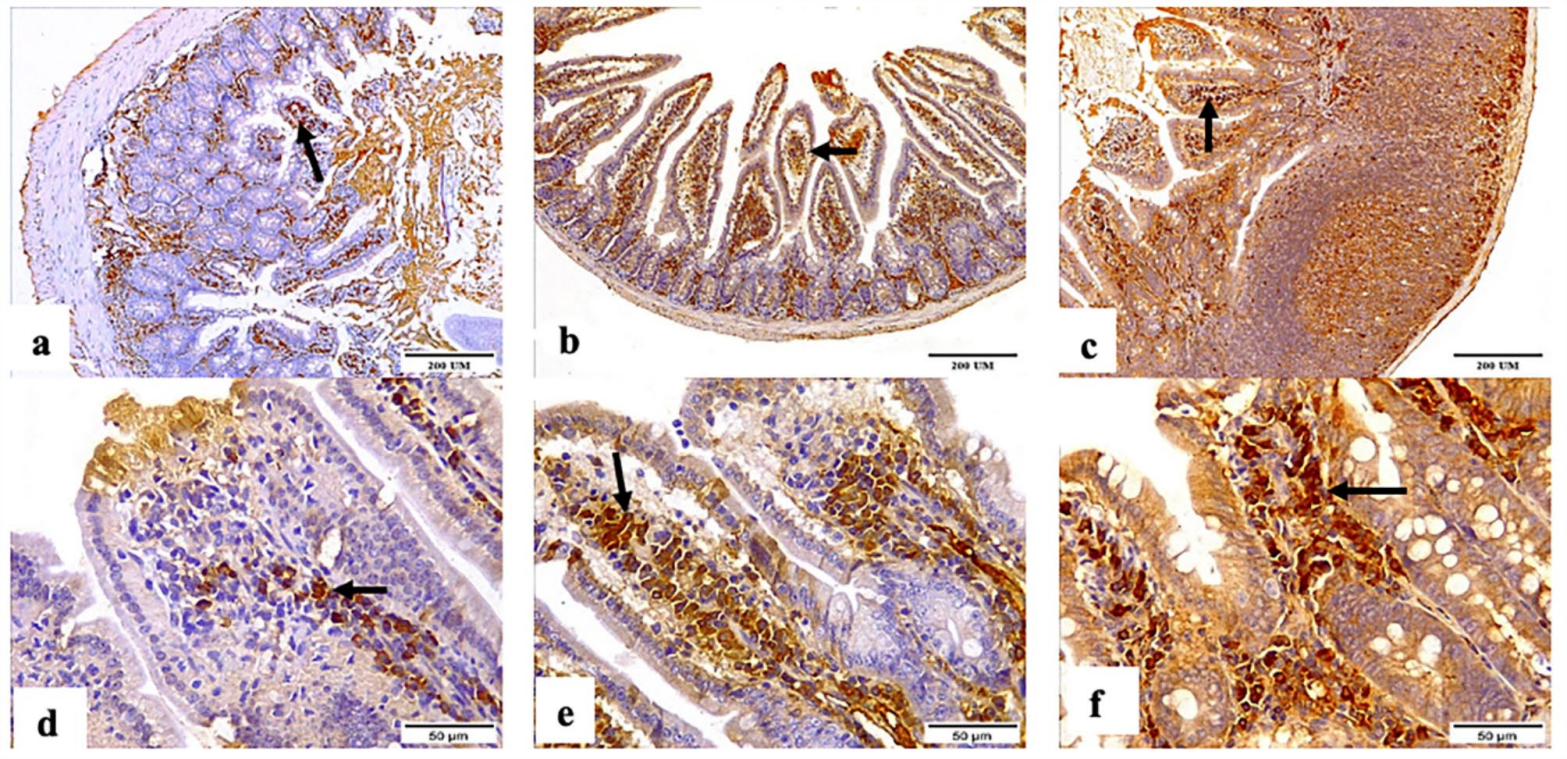
Photomicrographs showing CD8^+^ antibody reactivity in the ileal tissue sections of (**a and d**) the negative control, (**b and e**) *C. parvum-*infected group, and (**c and f**) immunized infected group indicating mild, moderate, and intense positive expression of CD8^+^ antibody, respectively (arrows). (CD8^+^ Antibody, magnification: 100 × and 400 ×, respectively, Scale bar: 200 and 50 μm, respectively)

**Fig. 4:**
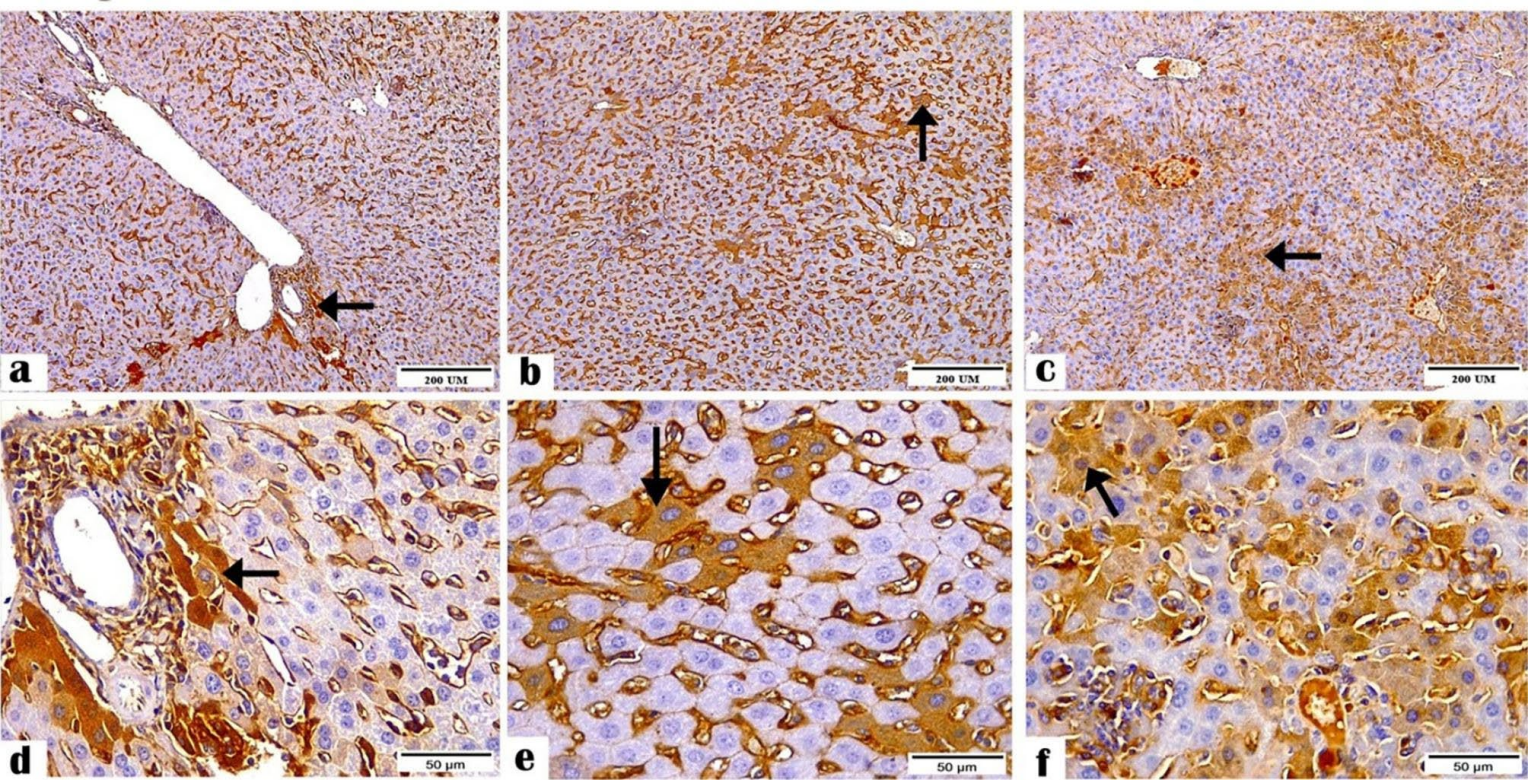
Photomicrographs showing CD8^+^ antibody expression in the liver tissue sections of (**a and d**) the negative control, (**b and e**) *C. parvum*-infected, and (**c and f**) immunized infected groups indicating mild, moderate, and strong positive expression of CD8^+^ antibody, respectively (arrows). (CD8^+^ Antibody, magnification: 100 × and 400 ×, respectively, scale bar: 200 and 50 μm, respectively)

The IHC results revealed the positive staining for CD4^+^ (Figs. 1 and 2) and CD8^+^ (Figs. 3 and 4) antibodies in the ileal and liver tissues of all groups. The negative control tissues, *C. parvum*-infected and the immunized infected groups showed mild, moderate, and high expression of CD4^+^ and CD8+, respectively.

**Fig. 5:**
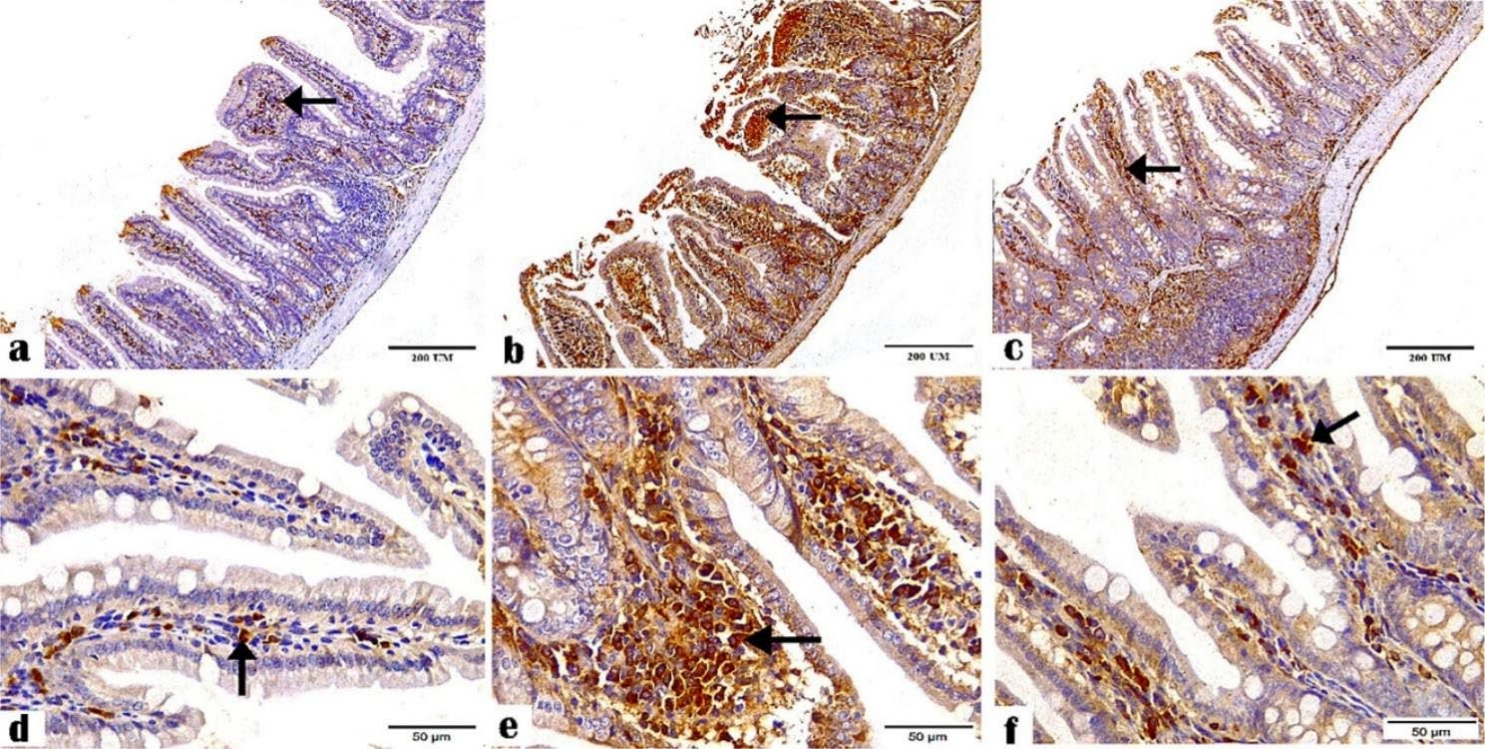
Photomicrographs displaying the Caspase-3 antibody expression in the ileal tissue sections of (**a and d**) the negative control, (**b and e**) *C. parvum*-infected, and (**c and f**) immunized infected groups indicating mild, highest, and moderate Caspase-3 antibody expression, respectively (arrows). (Caspase-3 Antibody, magnification: 10 × and 40 ×, respectively, scale bar: 200 and 50 μm, respectively)

Figure 5 showed the immune response of the ileal tissue sections to Caspase-3 antibodies in all groups. The negative control group showed mild Caspase-3 antibody expression, while the *C. parvum*-infected group exhibited the highest Caspase-3 reactivity among the examined groups. The immunized group showed moderate expression.

**Fig. 6:**
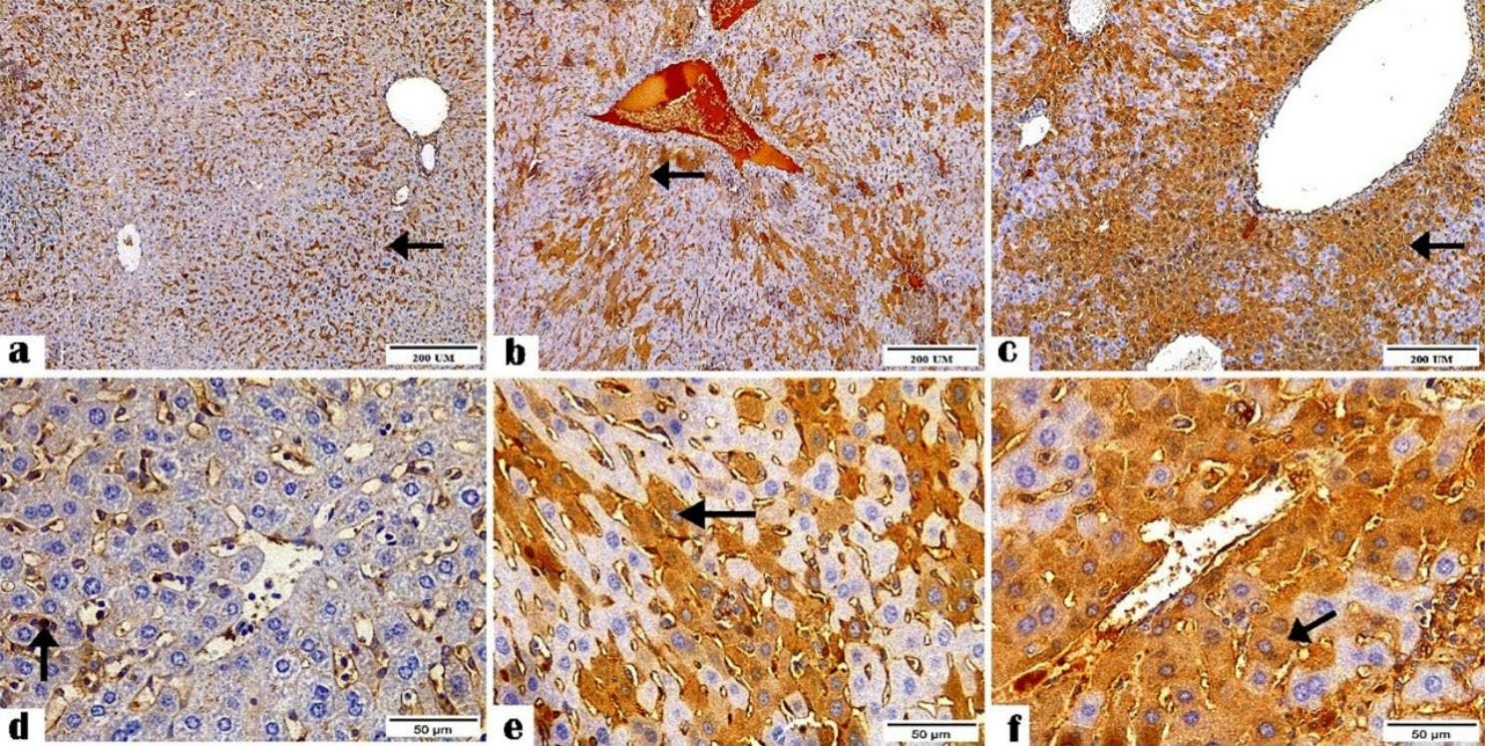
Photomicrographs showing the Caspase-3 antibody expression in the liver tissue sections of the (**a and d**) negative control, (**b and e**) *C. parvum*-infected, and (**c and f**) immunized infected groups showing scarce, moderate, and highest reactivity to the Caspase-3 antibody, respectively (arrows). (Caspase-3 Antibody, magnification: 10 × and 40 ×, respectively, Scale bar: 200 and 50 μm, respectively)

The liver tissue sections of the negative control and *C. parvum*-infected groups exhibited mild and moderate Caspase-3 expression, respectively (Fig. 6), while those in the immunized group showed the highest Caspase-3 antibody expression.

**Fig. 7:**
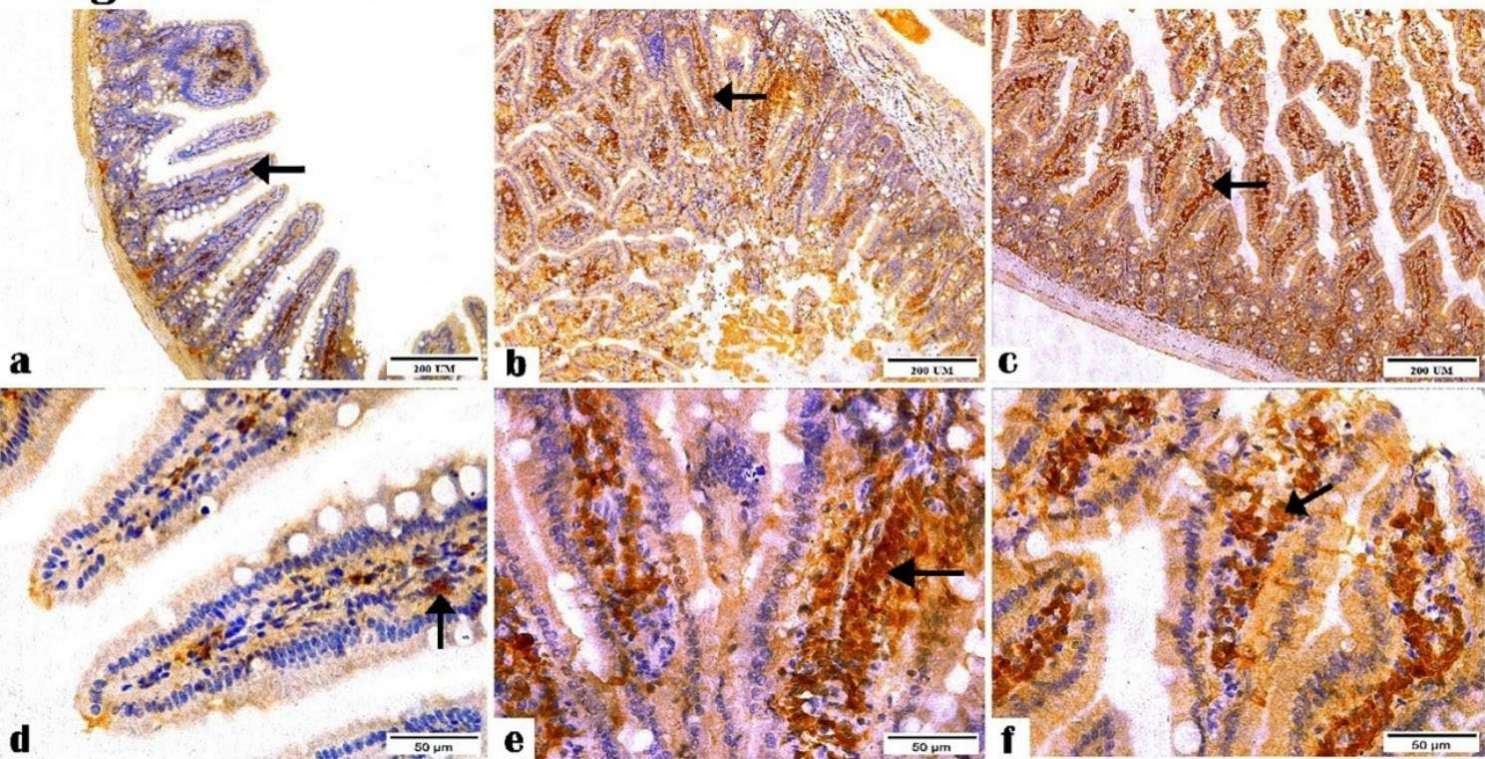
Photomicrographs showing the NF-κB antibody expression in the ileal tissue sections of the (**a and d**) negative control, (**b and e**) *C. parvum*-infected, and (**c and f**) immunized infected groups presenting mild, moderate, and highest reactivity to NF-κB antibodies, respectively (arrows). (NF-κB Antibody, magnification: 10 × and 40 ×, respectively, Scale bar; 200 and 50 μm, respectively)

**Fig. 8:**
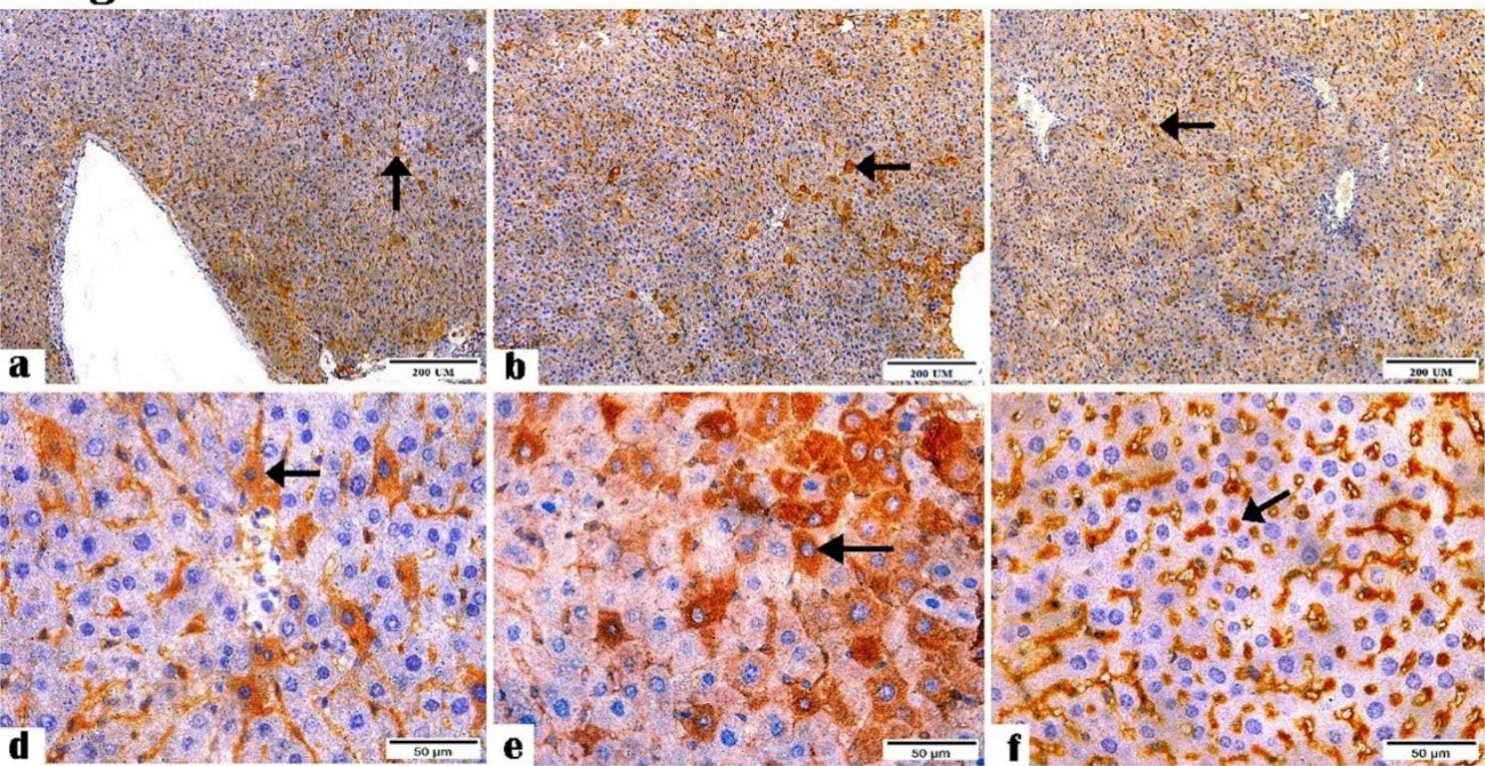
Photomicrographs showing NF-κB antibody expression in the liver tissue sections of the (**a and d**) negative control, (**b and e**) *C. parvum*-infected, and (**c and f**) immunized infected groups indicating mild cytoplasmic, moderate cytoplasmic, and highest nuclear reactivity to NF-κB antibodies (arrows). (NF-κB Antibody, Magnification: 10 × and 40 ×, respectively, Scale bar; 200 and

Figures 7 and 8 showed the immune reactivity of the ileal and liver tissue sections to NF-κB antibody. The sections of the negative control and *C. parvum*-infected groups displayed mild and moderate reactivity to NF-κB antibody, while the immunized infected group showed the highest reactivity.

### Immune-scoring area % to CD4^+^, CD8^+^, Caspase-3, and NF-κB

The immune-scoring results for CD4^+^, CD8^+^, Caspase-3, and NF-κB antibodies indicated a significant difference (*P <* 0.001) between the tissue sections of all groups (Tables 1 and 2). We also noticed high CD4^+^ and low CD8^+^ expression levels in the non-infected non-immunized group, while the opposite was seen in the infected non-immunized mice. In the immunized infected mice, the CD4^+^ levels were back to being higher than CD8^+^ in both organs.

**Table 1:** Immune-scoring area % to CD 4, CD 8, Caspase-3, and NF-κB in the ileal tissues of the examined groups

Antibody	Mean ± SE			F Value	*P* Value
	Negative control group	*C. parvum-*infected group	Immunized infected group		
CD 4	15.581 ± 0.35^c^	35.494 ± 0.55 ^b^	46.593 ± 0.61^a^	930.019**	0.000^**^
CD 8	13.394 ± 0.75^c^	37.44 ± 0.34^b^	43.769 ± 1.02^a^	445.933**	0.000**
Caspase-3	4.132 ± 0.293^c^	33.666 ± 1.413^a^	13.222 ± 0.495^b^	294.934	0.000**
NF-κB	5.4 ± 0.562^c^	49.558 ± 0.931^a^	44.269 ± 1.283^b^	616.424	0.000**

Values are expressed as Mean ± Standard Error. **P < 0.001 are highly significant

Different superscripts within the same row indicate significant differences

**Table 2:** Immune-scoring area % to CD 4, CD 8, Caspase-3, and NF-κB in the liver tissues of the examined groups

Antibody	Mean ± SE			F Value	*P* Value
	Negative control group	*C. parvum-*infected group	Immunized infected group		
CD 4	20.228 ± 0.65^c^	39.19 ± 0.81^b^	57.842 ± 0.69^a^	685.412**	0.000^**^
CD 8	17.824 ± 0.59^c^	40.04 ± 0.64^b^	52.603 ± 1.22^a^	411.784**	0.000^**^
Caspase-3	7.222 ± 0.522^c^	51.399 ± 0.924^b^	69.206 ± 1.353^a^	1033.556	0.000^**^
NF-κB	14.23 ± 0.65^c^	50.9 ± 0.868^a^	42.098 ± 0.75^b^	632.142	0.000^**^

Values are expressed as Mean ± Standard Error. **P < 0.001 are highly significant

Different superscripts within the same row indicate significant differences

Caspase-3 expression was higher in the ileal tissues of *C. parvum*-infected mice than in the immunized infected mice. Contrarily, it was higher in the liver tissues of immunized infected mice than in the *C. parvum*-infected tissues. NF-κB expression was higher in the *C. parvum*-infected ileal and liver tissues than in the immunized infected tissues.

## Discussion

The affinity-purified *C. parvum* oocyst antigen used in our study was previously characterized by SDS-PAGE, Western blot, and amino acid analysis, and its diagnostic potential was evaluated by ELISA [31]. As a vaccine candidate, it successfully imparted protective cellular and humoral responses against *C. parvum* in mice, significantly decreasing oocyst shedding, histopathological changes, and parasite developmental stages in the tissues [32].

Here, we chose the ileum as it is most affected by cryptosporidiosis in mice [33] and the liver as it is continuously exposed to many environmental pathogens and stress [34]. We used IHC staining to detect CD4^+^, CD 8, Caspase-3, and NF-κB as markers for T helper cells, cytotoxic T cells, apoptosis, and inflammation, respectively, in healthy, *C. parvum*-infected, and immunized infected mice groups. The immune-scoring program results verified a significant difference (*P <* 0.001) in CD4^+^, CD8^+^, Caspase-3, and NF-κB expression i in both ileum and liver tissues of all the groups, which might be important for immunomodulation in these regions.

Regarding the immune-scoring percentage, we reported high CD4^+^ and low CD8^+^ expression in the non-infected non-immunized groups. This was consistent with Fahmy et al. [35], who found that healthy non-infected mice exhibited a higher intestinal expression of local CD4^+^ T cells than CD8^+^ Tcells. Tauschmann et al. [36] found high CD4^+^ and low CD8^+^ T-cell counts in the small and large intestines. Several experimental studies on *Cryptosporidium* have linked the cell-mediated immune responses, especially the CD4^+^ T count, to the susceptibility to infection and severity of the disease. Although CD4^+^ T cells and IFN-γ are known to be essential for the clearance of *Cryptosporidium* infection, CD8^+^ T cells and humoral responses might also be involved [8]. The severity of cryptosporidiosis depends on the extent of immunosuppression, as indicated by the CD4^+^ T count [37]. A balanced CD4^+^/CD8^+^ T cell ratio is important for evaluating the immunomodulation response and the homeostasis of the immune system [38]. Here, we observed the reverse as we found that more CD8 + cells than CD4^+^ T cells in the infected non-immunized mice. However, Fahmy et al. [35] demonstrated overexpression of CD8^+^ T over CD4^+^ T cells in the *C. parvum*-infected mice intestines, which reversed the balanced CD4^+^/CD8^+^ ratio. The *C. parvum*-infected mice exhibiting high CD8^+^ expression did not show improvement in either parasite load or pathological consequences, as the CD4^+^ and CD8^+^ T cells were observed on the 10th day of infection (when the oocysts shedding, and pathological lesions were high). This suggests that inadequate CD8^+^ T cells might hinder the elimination of cryptosporidiosis in murine models [39]. We observed high expression of both CD4^+^ and CD8^+^ in immunized infected mice, where the CD4^+^ counts were back to being higher than CD8^+^ in both organs. This finding, combined with lower oocyst shedding and pathological lesions in the immunized infected mice than the infected non-immunized ones on the 10th day post-infection (as previously shown by our study: Aboelsoued et al. [32], might suggest the protective effect of the vaccine candidate.

Tissue damage at parasitic infection sites indicates host cell death or apoptosis in response to an intracellular pathogen, such as *C. parvum* [18, 19]. Infected host cells might be eliminated by activating caspases and inducing apoptosis to limit the spread of infection [40]. We observed mildly positive Caspase-3 expression in non-immunized non-infected mice tissues, consistent with Samaka et al. [41], who found low Caspase immunostaining levels in the absorptive epithelial cells. Also, we found that the immune scoring of Caspase-3 was higher in the ileal tissue of *C. parvum*-infected than in non-infected tissues, indicating signs of infection-induced apoptosis. These findings agreed with Sasahara et al. [42], who reported apoptosis in the ileum of neonatal mice after *C. parvum* infection and assumed that epithelial apoptosis might be integral for *C. parvum* pathogenesis. Moreover, Buret et al. [43] found that experimental *C. andersoni*, infecting human and bovine epithelial cells, can promote host cell apoptosis. Host cell apoptosis was also reported due to infection with *Trypanosoma cruzi*, *Toxoplasma gondii*, *Eimeria tenella*, and *Leishmania*, which is considered a host defense strategy [29, 44, 45].

Here, we observed moderate Caspase-3 expression in the ileal tissues of the immunized infected group, which was less than in *C. parvum*-infected non-immunized tissues, indicating the protective effect of our vaccine against apoptosis. Contrastingly, the Caspase-3 expression was higher in the liver tissues of immunized infected mice than in *C. parvum*-infected tissues, probably due to the stress associated with the Freund’s adjuvant used. However, this adjuvant might improve the effect of immunization in experiments and veterinary medicine as it initiates oxidative stress and inflammation in mice [46] and produces a strong and long-lasting inflammatory reaction [47]. Therefore, mixing vaccines with adjuvants enhances antigen-specific immunity, producing reactive oxygen species, which leads to apoptosis through factors such as Caspase-3 activation [48].

Mele et al. [25] showed the activation of apoptosis at the sporozoite and merozoite stages and inhibition of host cell apoptosis at the trophozoite stage after *C. parvum* infection in vitro, suggesting that the parasite might interact with host cells and regulate its gene expression. This supports the assumption that the modulation of the host cell apoptosis depends on the *Cryptosporidium* stage [49], as *C. parvum*-infected cells show signs of apoptosis shortly after the invasion and during the late infection stages. However, significant blockage of the apoptotic response is accompanied by parasite replication within the infected cells [25]. The *C. parvum* infection is considered to interfere with the temporal progression of the normal apoptotic response by delaying it sufficiently to help parasite replication. When the parasite becomes ready to exit the cell after replication, it either induces apoptosis or allows the cell to continue along its path, which is initiated during the early infection phases [24, 25].

We found that the NF-κB expression was higher in the ileum and liver tissues of *C. parvum*-infected mice than in non-infected ones, which might be supported by the hypothesis that *C. parvum* can neutralize the immediate cellular apoptosis response to sustain the infection and allow life cycle progression [18, 24]. Also, the inhibition of NF-κB enhanced *C. parvum*-induced apoptosis was observed in intestinal epithelial cells in vitro [18]. Furthermore, NF-κB activation-related protection against apoptosis in the infected cells had also been shown in other apicomplexan parasitic infections [29], such as *Toxoplasma gondii* [50] and *Theileria parva* [51] suggesting that this is possibly a common characteristic of these parasites.

In this study, NF-κB expression was lower in the immunized *C. parvum*-infected ileal and liver mice tissues than in the infected mice tissues, which might indicate the protective effect of our candidate vaccine against inflammation. This is consistent with our previous study, where we showed that immunized *C. parvum*-infected tissues displayed a slight pathogenic effect than non-immunized infected tissues [32]. This also agreed with Shang et al. [52], who found that vaccination suppressed NF-κB expression in the mucosal epithelium tissues and hence, blocked the inflammatory response. This vaccine-induced inhibitory mechanism might indicate how epithelium responds differentially to new or previously encountered pathogens. We showed a higher NF-κB profile in *C. parvum*-infected ileal and liver mice tissues than immunized infected mice tissues, measured as area % in a standard measuring frame Leica scoring program. This contradicts the IHC staining photomicrographs (Figs. 7 and 8) in which NF-κB expression was higher in the ileal and liver tissues of immunized infected mice than in *C. parvum*-infected mice tissues, indicating a possible nonspecific reaction in the *C. parvum*-infected tissues.

## Conclusion

Developing effective vaccines against cryptosporidiosis requires a better understanding of both the parasite and host immune responses. Our IHC staining results, evaluated using an immune-scoring program, showed that CD4^+^, CD8^+^, Caspase-3, and NF-κB profiles might be important for immunomodulation in the ileal and liver tissues. We observed improvement in immunized infected mice, indicating that the prepared vaccine candidate might effectively protect against cryptosporidiosis. Further studies using different animal hosts and adjuvants are warranted for getting a deep understanding the efficacy of this vaccine at the molecular level.

## Materials and methods

### Animals

#### Mice

We obtained seven pregnant laboratory-bred Swiss albino mice from the animal house at the National Research Centre, Egypt, and adapted them to the experimental conditions for a week. All the pregnant mice were confirmed to be parasite-free by fecal examination for three consecutive days using flotation, sedimentation and modified Ziehl Neelsen (MZN) techniques [53] before starting experiments. The experimental area was strictly sanitized. Food and water were provided *ad libitum*. On day 3 after birth, 30 newborn mice were randomly divided into groups for experiments.

#### Rabbits

We used five male New Zealand rabbits aging six weeks and weighing 1.5–2 kg. The rabbits were purchased from local poultry markets and we cannot obtained informed consent from the owner. These rabbits were confirmed to be parasite-free by fecal examination for three consecutive days using flotation, sedimentation and MZN techniques [53] before starting experiments. After adapting to the experimental conditions for a week, the animals were given food and water *ad libitum* under strict, sanitized conditions.

#### Parasite

We used the *C. parvum* isolate (GenBank ON730707) that was previously identified using the *Cryptosporidium* oocyst wall protein (COWP) gene by PCR from fecal samples collected from ten newborn buffalo calves (aging 10–20 days) reared by local farmers in Beni-Suef Governorate, Egypt [32]. The authors confirm that permission from the farmer was obtained for collecting fecal samples from newborn buffalo calves.

### Experimental design

#### Propagation of oocysts

To produce more oocysts for preparing the *C. parvum* oocysts antigen, ten parasite-free neonatal Swiss albino mice (three days of age) were experimentally infected using a single dose of 1 × 10^5^* C. parvum* oocysts collected from naturally infected buffalo calves using gastric tubes 1 h before a meal [54]. After three days, the fecal pellets were collected daily for three weeks and examined using the MZN technique [53]. Then, the oocysts were concentrated by centrifuging (500 ^×^g) using Sheather’s sugar solution [55] and preserved in potassium dichromate solution (2.5%) at 4^°^C. Before experimental infection into mice, they were washed by phosphate buffered saline (0.01 M, pH 7.2), concentrated, and counted using a hemocytometer [56].

#### Preparation of oocysts antigen

*C. parvum* oocyst antigen was prepared by sonication (Sonics Vibra-Cell VCX750 USA; 12 cycles/30 seconds and then centrifuged at 12,000 ×g for 15 min at 4^°^C) after 20 freeze-thaw cycles according to Aboelsoued et al. [57].

#### Preparation of rabbit hyperimmune serum

The rabbit hyperimmune serum against the *C. parvum* oocyst antigen was prepared according to Fagbemi et al. [58]. After collecting the blood samples from the rabbit’s ear vein, the sera were separated and stored at − 20^°^C until further use. After preparation of the hyperimmune serum in the experiment, the rabbits used were not euthanized and released.

#### Affinity purification of *C. parvum* oocyst soluble antigen

The prepared rabbit hyperimmune serum was defrosted and dialyzed for three days in a coupling buffer and then coupled to cyanogen bromide-activated Sepharose-4B (Sigma Aldrich) (2 mg/ml-swollen beads) according to the manufacturer’s protocol. The *C. parvum* oocyst antigen was applied to the affinity column (Flex-Column, Kimble, USA) as described by Toaleb et al. [59]. The bound fraction was eluted using the elution buffer. The protein content of the purified antigen was estimated using the Lowry’s method [60]. and stored at − 20^°^C until use.

#### Mice immunization

The parasite-free pregnant laboratory-bred Swiss albino mice were obtained and adapted to the experimental conditions for a week. Three-day-old newborn mice were randomly divided into three groups (10 mice/group). The first group was the negative control, and the 2nd group (positive control) was experimentally infected orally with a dose of 1 × 10^5^ isolated *C. parvum* oocysts in 250 µl PBS solution (pH 7.2) [54] using gastric tubes 1 h before a meal. In the 3rd group, the mice were injected subcutaneously with 40 µg/kg of the *C. parvum* purified fraction (as a vaccine) emulsified in complete Freund’s adjuvant for primary immunization [61]. After two weeks, a booster dose containing another 40 µg/kg of the same purified fraction in incomplete Freund’s adjuvant was administered. Two weeks after the booster each mouse was infected orally by 1 × 10^5^* C. parvum* oocysts, and the positive control group was experimentally infected simultaneously. All mice were euthanized on the 10th day post-infection (at the peak of oocysts shedding) by cervical dislocation under anesthesia with using intra-peritoneal injection of sodium pentobarbital at dose of 40 to 50 mg/kg [32]. The small intestine (ileum) and liver tissues were collected from all the mice and fixed in 10% neutral buffered formalin for 24 h. Then, they were trimmed, washed, dehydrated, cleared in xylene, and embedded in paraffin wax, as described by Bancroft and Stevens [62].

#### Immunohistochemistry (IHC) staining

IHC was performed using the paraffin sections mounted on positively charged slides to detect CD4^+^, CD8^+^, Caspase-3, and NF-κB expression as indicators of T helper cells, cytotoxic T cells, apoptosis, and inflammation using the avidin-biotin-peroxidase complex (ABC) method [63]. Polyclonal anti-CD4^+^ (1:50–1:200, Quartett, Cat# CD024-02), monoclonal anti-CD8^+^ antibody (C8/144B, 1:25–1:100, Quartett, Cat# CD039-10), polyclonal antiactive Caspase-3 (cleaved form, 1:50–1:100, Sigma Diagnostic Biosystems, Cat# RP096) and monoclonal anti-NF-κB2 (Sigma Aldrich, Cat# AMAB91511) were used. The ileum and liver sections from each group were incubated with the above mentioned antibodies, and the reagents required for the ABC method (Vectastain ABC-HRP kit, Vector laboratories) were added. Marker expression was labeled with peroxidase and colored with diaminobenzidine (DAB, Sigma) to detect the antigen-antibody complex. The negative control consisted of a nonimmune serum instead of primary or secondary antibodies. The stained sections were examined under a microscope (CH9435 Hee56rbrugg, Leica Microsystems, Switzerland).

#### Evaluation of IHC results in terms of “area percentage” (specific area/Antibody)

The areas displaying positive brown immunostaining were evaluated regardless of the staining intensity using some features (cell counter, color deconvolution, color threshold, and IHC plugin) of the Leica scoring program. The measurement units (pixels) were converted into actual micrometer units. CD4^+^, CD8^+^, Caspase-3, and NF-κB immunostaining were measured as area % in a standard measuring frame, representing six fields for each subject (ileum and liver) in all groups under 400^x^ magnification.

### Statistical analysis

Statistical analysis was performed with SPSS 19.0 for Windows (Statistical Package for Scientific Studies, SPSS, Inc., USA). The values were represented as mean and standard error. The immune-scoring data was tested for normality via the Kolmogorov-Smirnov test of normality, whose outcomes indicated that the highest data were normally distributed (parametric data). Thus, one way-analysis of variance (ANOVA) and post-hoc tests were used to compare the groups. Values with *P <* 0.05 were considered statistically significant.

## Data Availability

The database used or analyzed during this study are available from the corresponding author on reasonable request.

## Abbreviations

*C. parvum*: *Cryptosporidium parvum*
*IHC*: Immunohistochemical
SPSS: Statistical Package for Social Sciences
*ANOVA*: Analysis of Variance
